# Long-distance continuous-variable quantum key distribution by controlling excess noise

**DOI:** 10.1038/srep19201

**Published:** 2016-01-13

**Authors:** Duan Huang, Peng Huang, Dakai Lin, Guihua Zeng

**Affiliations:** 1 State Key Laboratory of Advanced Optical Communication Systems and Networks, and Center of Quantum Information Sensing and Processing, Shanghai Jiao Tong University, Shanghai 200240, China; 2 College of Information Science and Technology, Northwest University, Xi’an 710127, Shaanxi, China

## Abstract

Quantum cryptography founded on the laws of physics could revolutionize the way in which communication information is protected. Significant progresses in long-distance quantum key distribution based on discrete variables have led to the secure quantum communication in real-world conditions being available. However, the alternative approach implemented with continuous variables has not yet reached the secure distance beyond 100 km. Here, we overcome the previous range limitation by controlling system excess noise and report such a long distance continuous-variable quantum key distribution experiment. Our result paves the road to the large-scale secure quantum communication with continuous variables and serves as a stepping stone in the quest for quantum network.

Quantum key distribution (QKD) using photons to disseminate encryption codes enables two distant partners to share a secret key^1^,^2^. Currently, two available approaches referred to as discrete-variable QKD^3^,^4^ and continuous-variable (CV) QKD^5^,^6^ are employed to distribute secret keys. The CV-QKD has been proved, in principle, to be secure against general collective eavesdropping attacks, which are optimal in both the asymptotic case^7^,^8^ and the finite-size regime^9^,^10^,^11^. From a practical point of view, the CV approach has potential advantages^12^ because it is compatible with the standard optical telecommunication technologies. It is foreseeable that this approach will become a viable candidate for large-scale secure quantum communication.

However, the practical long-distance environment provides a number of technical challenges for the present CV-QKD experiments. There are two major hurdles that severely limit the secure distance. One is limited reconciliation efficiency^13^ and the other is excess noise^14^. Because of the difficulty of reconciliation at low signal-to-noise ratios (SNRs) in previous 25 km experiments^15^,^16^,^17^,^18^,^19^, P. Jouguet *et al.* developed an efficient error-correcting code (ECC)^20^ which leads to a remarkable improvement of transmission distance for CV-QKD^21^. However, further extending the secure distance in their experiment is limited, partly because of the incremental technical excess noise. Intuitively, the increase of excess noise is associated with higher fibre loss and lower SNR, which are two key aspects of long-distance implementations. More specifically, the increase of fibre loss requires a stronger Local Oscillator (LO) for shot-noise-limited homodyne detection, and the decrease of SNR is a great challenge for precision phase compensation. Nevertheless, in the long-distance scenarios, control of the excess noise induced by the photons leakage from the strong LO to the weak quantum signal and the inaccuracy of phase compensation has never been studied experimentally. This may be attributed to the fact that beyond 100 km the experimental difficulties of CV-QKD are significantly increased with respect to previous achievements.

In this paper, we report for the first time an experimental demonstration of CV-QKD over 100 km fiber channel. The result is achieved by controlling the excess noise in the following ways. Firstly, the adoption of a high-sensitive homodyne detector with lower requirement of LO power allows us to reach the shot noise limit (SNL) at previously inaccessible parameter regions, and it is the prerequisite of successfully performing a long-distance CV-QKD experiment. Secondly, a secure scheme is proposed to overcome the difficulty of high-precision phase compensation under the low SNR conditions, so that we can get the effective data regardless of the phase drifts of fibre links. Both techniques confine the excess noise within a tolerable limit, and result in a record secure transmission distance. Another practical distance limitation of our experiment is essentially the finite-size effect, and appear to be due mostly to the excess noise induced by finite statistics^9^,^10^,^11^. However, the key element for the present experiment is controlling the technical excess noise that is previously overlooked, and we verify the applicability and maturity of such technologies in real-world scenarios.

## Results

### Experimental setup

**Figure 1:**
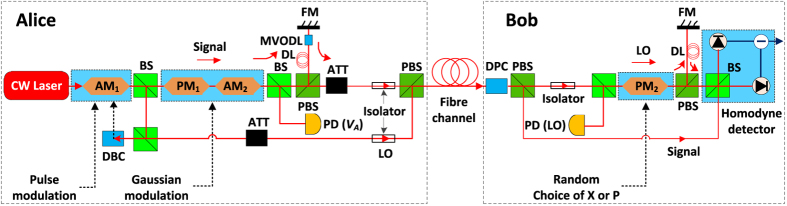
Experimental setup of CV-QKD. CW laser, continuous wave laser; AM, amplitude modulator; DBC, dynamic bias controller; BS, beam splitter; ATT, attenuator; PM, phase modulator; PD, photodetector; PBS, polarizing beamsplitter; DL, delay line; MVODL, manual variable optical delay line; FM, faraday mirror; DPC, dynamic polarization controller.

We perform the experiment based on the Gaussian-modulated coherent states (GMCS) protocol^12^. The experiment setup is depicted in Fig. 1. It consists of three major steps: pulse modulation, Gaussian modulation and random phase modulation for homodyne detection. At Alice’s side, a 1,550 nm continuous-wave (CW) light is transformed into a 2 MHz clock square pulse train by an amplitude modulator (AM) in pulse modulation. An asymmetrical Mach-Zehnder interferometer (AMZI) divides the pulses into a LO path and a signal path. In the signal path, the *x* and *p* quadratures of coherent states are modulated in according to a centered Gaussian distribution of variance *V*_*A*_ in the units of shot noise variance *N*_0_, where *N*_0_ appears in the Heisenberg uncertainty relation 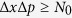. By using the polarization-multiplexing and time-multiplexing techniques, the signal together with LO are sent to Bob through a 100 km standard telecom fiber spool with a measured loss of 0.2 dB/km at 1,550 nm. For the polarization-multiplexing, the Faraday mirrors reflects the signal pulses at Alice’s side and LO pulses at Bob’s side by imposing a 90° rotation on their original polarization states. Besides, two delay lines and a manual variable optical delay line are inserted into the system so as to accurately equilibrate the interferometer. At Bob’s side, the demultiplexed LO and signal interfere in a shot-noise-limited homodyne detector. The output intensity is proportional to the modulated quadratures. Bob measures either *x* or *p* by randomly generating a *π*/2 or zero phase shift on the reference LO light. To enhance the system stability, we developed automatic feedback modules to calibrate the bias of AM at Alice’s side and the polarization-demultiplexing at Bob’s side (see Methods and Supplementary for more details).

In our experimental setup, we insert several isolators in both sides to prevent the Trojan-horse attacks^22^. Since the shot noise variance is proportional to the LO power, we use a photodiode (PD) to monitor the LO at Bob’s side which is transmitted through the insecure quantum channel and it could be manipulated by a potential eavesdropper. In addition, a recent robust shot noise measurement scheme^23^ can also be employed in our experiment to prevent some attacks targeting the shot noise, such as LO fluctuation attacks^24^ and LO calibration attacks^25^,^26^. The potential risk of other attacks can also be resisted by additionally inserting optical devices. For example, the wavelength attacks^27^ can be prevented with a fiber Bragg grating at Bob’s side. In the following, we employ the general assumption that Eve cannot tamper with the devices in both sides. In this case, the detection efficiency *η*_*hom*_ and the electronic noise *υ*_*el*_ can be considered to be inaccessible to Eve. The other experimental parameters associated with the secure distance, such as *V*_*A*_, *N*_0_, channel transmission *T* and excess noise *ε*, are estimated using a parameter estimation process in real time, where *ε* and *υ*_*el*_ are expressed in shot noise units.

### Controlling excess noise by shot-noise-limited homodyne detection with weak LO

In the implementation of the GMCS protocol, homodyne detection of coherent states under the SNL requires sufficient LO power. However, the excess noise increases significantly due to photons leakage from the strong LO to the weak quantum signal. Especially, in an optical system with a finite extinction ratio *R*_*e*_, it is difficult to completely remove the residual photons between two adjacent LO pulses. Since the leaked LO photons and the signal photons will simultaneously interfere with the LO pulses, the excess noise *ε*_*LE*_ and *V*_*A*_ would be of the same order of magnitude. The involved excess noise *ε*_*LE*_ is derived in Supplementary S1,


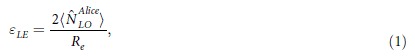


where 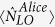 is the LO power at Alice’s side. In Fig. 1, we achieved an overall equivalent extinction ratio of 100 dB with customized optics which feature an extinction ratio of 65 dB in pulse modulation and 35 dB in polarization-multiplexing. In the previous experiments^15^,^16^, the typical LO power 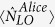 is 10^8^~10^9^ photons/pulse. In order to achieve a secure distance of 100~150 km (or equivalently 20~30 dB fiber loss), the tolerable excess noise is around 0.01 under the collective attacks. Therefore, at Bob’s side, the shot-noise-limited homodyne detection should be performed with a weak LO 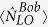 of 10^5^ photons/pulse. However, to our knowledge, the reported state-of-art shot-noise-limited homodyne detector is usually operated at a LO power 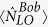 of 10^6^ ~ 10^8^ photons/pulse^28^, and this quantum detector has been widely used in the shot-noise-limited measurement of quantum state^29^,^30^,^31^.

To understand that the insufficient LO power 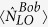 for the shot-noise-limited homodyne detection has become a major constraint in the long-distance CV-QKD, it is useful to start with analysis of shot noise *N*_0_ and electronic noise *υ*_*el*_ in terms of the noise ratio


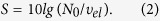


The basic prerequisite of the shot-noise-limited homodyne detection is *S* > 0 dB. And the shot noise *N*_0_ is associated with the LO power 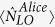 at Alice’s side,


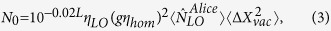


where *L* is the fibre length, *η*_*LO*_ is the LO transmittance at Bob’s side, *g* is the electronic gain, and 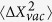 is the vacuum fluctuation. According to Eqs (2) and (3), it is clear that the homodyne detection in the SNL requires relatively low electronic noise and sufficient LO power at Bob’s side, whereas the latter is limited by the maximum tolerable excess noise *ε*_*LE*_ in 100 ~ 150 km CV-QKD as discussed above.

**Figure 2:**
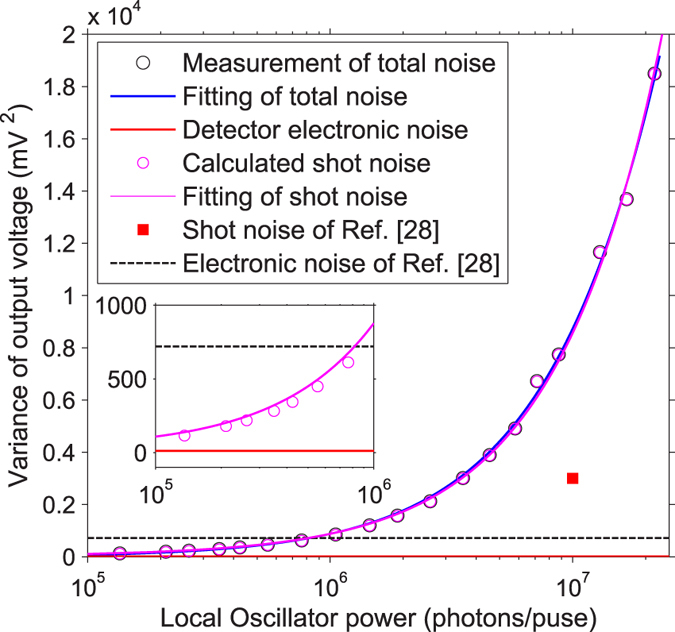
Shot noise characterization of homodyne detector. (**1**) The total noise (black circles) includes: (i) shot noise *N*_0_ (linear LO-dependent), (ii) electronic noise 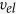 (LO-independent) and (iii) the noise of LO fluctuations 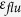 (quadratic LO-dependent). The measurement of total noise is fitted by a quadratic polynomial function with confidence intervals of 0.95 (blue line). The error bars are much smaller than the symbol size. (**2**) The electronic noise 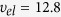 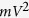 (red line) is measured without LO. (**3**) The shot noise (magenta circles) is calculated from the measurement total noise. The calculated shot noise is fitted by a linear polynomial function with confidence intervals of 0.95 (magenta line), which can be written as *N*_0_ = 8.5 × 10^−4^ 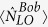 ≈ 8.5 × 
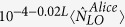
.

To control the excess noise to a level that makes the long-distance experiment possible, we developed an extremely low electronic noise homodyne detector, which allow us to reach the SNL with a weak LO. We replace the conventional operational preamplifier with cooled field-effect transistors (FETs) because that the FETs have been shown superior performance in low-noise applications. The additional cooling increases the transconductance and reduces the leakage current, subsequently reduces the electronic noise. The FETs employed in our detector are fabricated in a multistage thermoelectric cooler with minimum temperature of −510 °C. We note that the FETs with the cryogenic operation have been used in the photon-number-resolving detection^32^. It has great advantage because the noise figure (NF) of first stage completely dominates the NF of the entire detector. In these ways, we designed a nearly noise-free preamplifier circuit. The overall detection efficiency *η*_*hom*_ is 0.6, which is limited by the quantum efficiency of PIN photodiodes. The total noise of the detector is measured by a 200 M/s data acquisition card with a 50 ns width pulsed LO at a repetition rate of 2 MHz. In Fig. 2, each noise variance point is obtained from 10^7^ sample pulses. One great benefit of handling the electronic noise *υ*_*el*_ is that we can achieve a large electronic gain coefficient *g* in our design so as to get higher noise clearance between shot noise and electronic noise compared with previous detector^28^. The achieved maximum noise ratio *S* is 30 dB, 19 dB, 8 dB at LO power of 10^7^, 10^6^, 10^5^ photons/pulse, respectively. In this way, with a typical 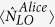 of 10^8^ photons/pulse and extinction ratio *R*_*e*_ of 100 dB, we achieved the noise ratio of *S* > 8 dB and effectively controlled the excess noise *ε*_*LE*_ in the order of 0.01, which is a tolerable value in our 100 ~ 150 km CV-QKD experiment.

### Controlling excess noise by high-precision phase compensation with low SNR

In the GMCS-QKD implementation, the phase difference *ϕ* between the LO (phase reference) and the quantum signal will drift with time due to the instabilities of AMZIs. Accordingly, a phase compensation scheme is necessary. However, under low SNR situations, the attempt to compensate the phase drift with stronger optical signals compared with the quantum signals, such as brighter labeling pulses, would leave a loophole for Eve. Moreover, the increase of inaccuracy *δθ* of phase compensation at low SNR will inevitably result in higher level of the excess noise. Here we developed a secure way to control the excess noise, and it is realized with a high-precision phase compensation by means of software based on noisy raw data, which is randomly selected from Gaussian raw keys in the postprocessing process.

We firstly characterize the phase drifts and the corresponding excess noise *ε*_*phase*_ in a CV-QKD experiment. The phase difference *ϕ* in one frame can be described as,


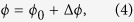


where *ϕ*_*0*_ is the relative phase difference (or the phase difference when Alice encodes phase 0) which is constant during one frame transmission, and Δ*ϕ* = *ϕ*_*max*_−*ϕ*_*min*_ is a small variation of the phase drift in one frame. Because the phase difference *ϕ* is the only estimated value for the phase compensation in the transmission period of one frame, in order to effectively compensate the phase drift, the phase variation Δ*ϕ* of the phase drift in this frame should be less than the inaccuracy *δθ,* i.e. the precision of the phase compensation. Otherwise, the phase difference between the real phase drift and compensate phase value might be exploited by a potential eavesdropper in this data frame, and the estimated excess noise would be lower than the actual value. Therefore, one has to achieve


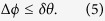


To confine the phase excess noise within a tolerable limit, we have derived *ε*_*phase*_ due to the inaccuracy of the phase compensation in Supplementary S1,


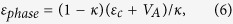


where 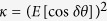, 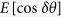 denotes the expectation of the cos*δθ*, and *ε*_*c*_ is the channel excess noise^33^. According to Eq. (6), to suppress the phase excess noise *ε*_*phase*_ to a level of 0.01 or 0.001 with typical values *ε*_*c*_ < 0.01 and *V*_*A*_ = 4, the minimum precision requirement of *δθ* is 2.9° or 0.9° per frame, respectively. In these two cases, according to Eq. (5), the variations of phase drifts Δ*ϕ* in one frame should be less than 2.9° or 0.9°, respectively.

Our phase compensation scheme is described as follows (see Methods). In the reconciliation stage Bob announces a randomly selected subset 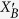 of one frame, and then Alice makes a reverse prediction of *ϕ* by calculating the auto-correlation of her original frame 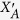 and Bob’s noisy detection results 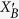,


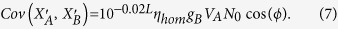


where *g*_*B*_ is the overall gain coefficient at Bob’s side. It is clear that the auto-correlation results are irrelevant to the Gaussian noise. This way enables us to compensate the phase drift *ϕ* under a low SNR condition.

**Figure 3:**
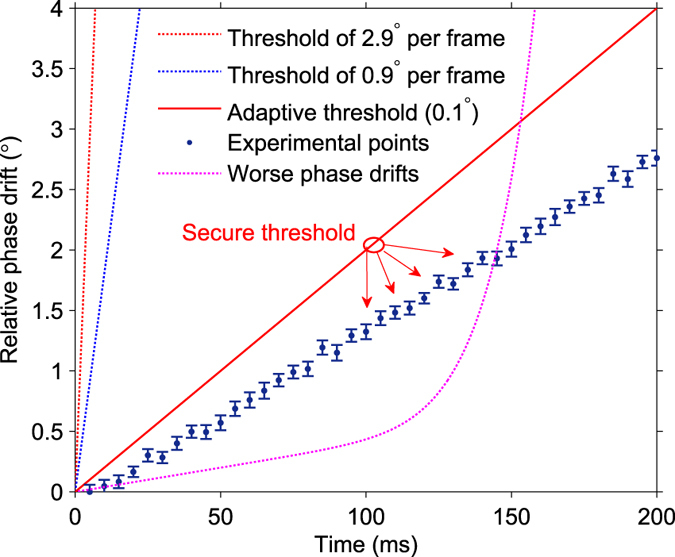
Characterization of phase compensation. The adaptive threshold is calculated from a subset of the raw data. The maximum length of error bar *L*_*E*_ represents the accuracy 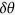 of the phase compensation, which is used to confine the excess noise 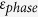. The slope of the adaptive threshold is determined by the 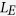 (°/frame, one frame is 5 ms in our case). The worse phase drifts will be confined by a straight line with a bigger slope, which means higher excess noise 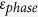. The total phase drifts is about 15°/s (<0.1°/5 ms) in our case.

The characterization of the phase compensation in our experiment is shown in Fig. 3. We use a secure threshold to estimate the excess noise *ε*_*phase*_ due to *δθ*. We firstly compute the phase drifts of measurement results in one data block. The maximum inaccuracy of the phase compensation in the block is used to set an adaptive threshold and bound the excess noise *ε*_*phase*_. In our case, each experiment point of phase drift *ϕ* is calculated with 4 × 10^3^ pulses which are randomly selected in one frame. The elapsed time of one frame is 5 ms which corresponds to 10^4^ pulses. The length of error bar represents the accuracy *δθ* of the phase compensation. The maximum *δθ* in one block (40 frames in our case) is used to set as the secure threshold, and the excess noise *ε*_*phase*_ is calculated based on the threshold. Under the normal condition, we achieved *ε*_*phase*_ < 1.2 × 10^−5^ with a typical SNR of ~0.002 and the compensation accuracy *δθ* of 0.1° per frame in the 150 km CV-QKD experiment. Under a worse condition, for example, a measurement block with complex and worse phase drifts can be divided into slow phase drifts ( < 100 ms) and fast phase drifts 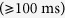, whereas the latter might be caused by Eve’s phase attacks. According to Eqs. (5) and (6), the portion of block with a tendency of phase drifts towards to Δ*ϕ* = 2.9° per frame will be discarded directly. While a randomly selected subset of the other portion will be used to compute a reliable adaptive threshold, which is used to calculated the maximum excess noise of phase compensation. Since this randomness makes it hard for Eve to guess the calculated pulses, we can guarantee the security of our phase compensation scheme in a CV-QKD experiment, and confine the phase excess noise within a tolerable limit.

### Reconciliation and finite-size secret key

In a 100~150 km CV-QKD implementation, the SNR is lower by more than an order of magnitude compared with previous record^21^. Hence it imposes a greater challenge to reconcile Gaussian variables. In our experiment, the actual SNRs at Bob’s side are about 0.024 at 100 km and 0.0024 at 150 km, which are achieved by optimizing the modulation variances *V*_*A*_ (detailed in Supplementary S2). Based on the multiedge-type low density parity check codes^34^ and the technique of repetition scheme^35^, we developed a 25 MHz ECC^36^ which exhibits an efficiency of *β* = 95.6% at the SNR threshold of 0.002 (detailed in Supplementary S2). The target frame error rate (FER) is 0.3. We remark here that the FER is one of key characteristics of an ECC. The failure probability of error decoding cannot be neglected and should be calculated in the final key rate. Taking the finite-size effects into account, the maximum secret key rate bounded by collective attacks is given by


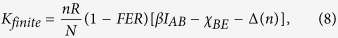


where *I*_AB_ is the Shannon mutual information between Alice and Bob, *χ*_BE_ is the Holevo bound on the information between Bob and Eve, *R* is the repetition rate of QKD system and it is 2 MHz in our experiment, Δ(*n*) is related to the security of the privacy amplification^9^, *N* denotes the sampling length, and 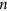 denotes the block length for final key estimation. In the post-processing procedure, the block with a length of *N*−*n* is used for parameters estimation and phase compensation, and *n/N* ≈ 2/7 in our case.

**Figure 4:**
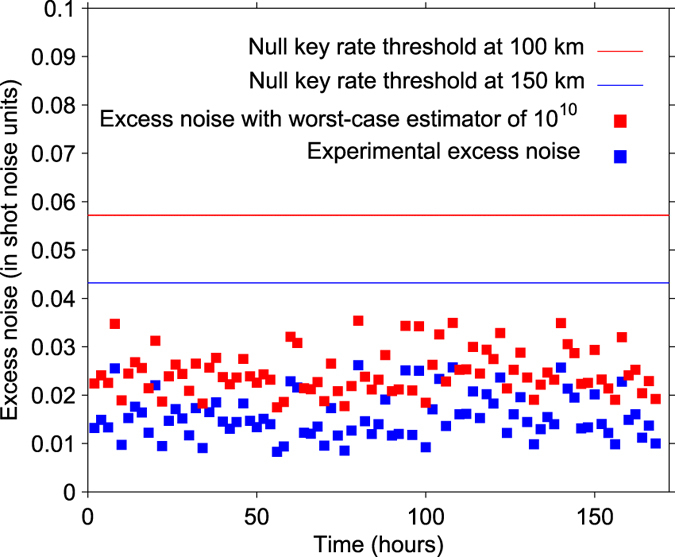
Excess noise measurement. The lower blue square points are measured at 100 km with 10^10^ finite-size blocks, which are subsets of a block with size of 10^12^. The effective excess noise under worst-case estimator (red square point) is employed to compute the final secret key rate. The red line defines the tolerable maximal value of excess noise at 150 km. The blue line defines the tolerable maximal value of excess noise at 100 km.

In Fig. 4, we mainly focus on the excess noise in the parameters estimation process. The excess noise is measured on a block of size 10^12^ with 84 blocks of size 10^10^ over one week for a distance of 100 km. Fortunately, because we have employed a high sensitive homodyne detector in weak LO and high precision phase compensation under low SNR condition, we can well deal with the main excess noise due to the inherent defects of the long-distance CV-QKD experiment. The measurement of the excess noise with the finite-size block of 10^12^ is around 0.015*N*_*0*_. The corresponding excess noise under the worst-case estimator^9^,^21^,^37^ is employed to compute the secret key rate when the extreme finite-size effects is taken into account.

**Figure 5:**
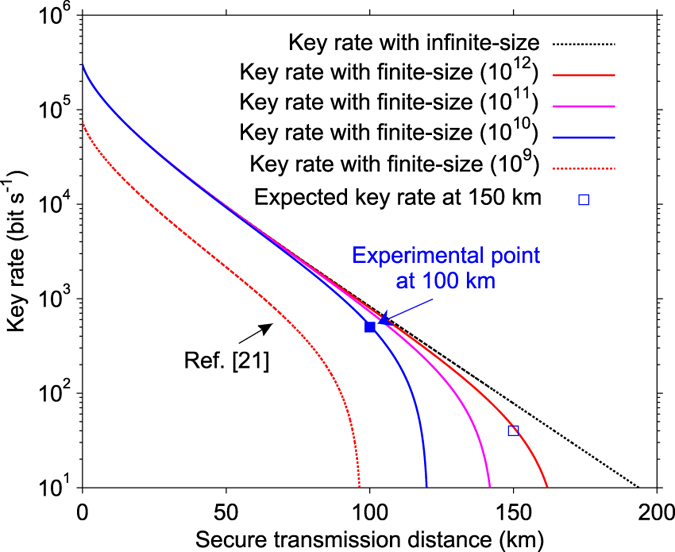
Secret key rate under general collective attacks in finite-size scenarios. The finite-size security model is based on the previous state-of-art experiment^21^. From left to right, curves correspond, respectively, to block lengths of 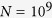,10^10^,10^11^,10^12^ and infinite. The red dash line is the state-of-art experiment with 1 MHz repetition rate. The blue square point is the 2 MHz experiment result, which is calculated from a group of time-varying excess noise. The modulation variance 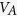 is optimized and set as 4, the reconciliation efficiency 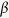 is 95.6%, the theoretical target excess noise is set as 0.01, the security parameter 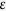 is set as 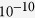. The practical excess noise with worst-case estimator is used to compute the experimental point.

The secret key rate respect to transmission distance is depicted in Fig. 5. The key experimental parameters that intervene in the Eq. (8) for the calculation of key rate are the modulation variances *V*_*A*_, channel transmission *T*, excess noise *ε*, the quantum efficiency *η*_*hom*_, and the electronic noise *υ*_*el*_, which are estimated in a finite-size scenario. Results show that we have experimentally achieved a secure transmission distance over 100 km. Based on the realistic experiment conditions, the maximum achievable block size in our experiment is 10^12^, which takes one week for the data acquisition and processing. For simplicity, we present one finite-size block with size of 10^12^ in Fig. 4 to demonstrate the ability to implement such a long-distance experiment. For comparison, we plot the previous state-of-art experimental result^21^ with 10^9^ finite-size block at the same finite-size security model^9^,^37^.

We also became aware of a recent work on finite-size effects for CV-QKD^11^. It shows a tighter security bound to describe Eve’s attacks in composable security framework, which requires larger blocks for parameter estimation. With such a finite-size security model, the minimum finite-size block is 10^9^ at 10 km. Fortunately, the security of our system with our excess noise controlling techniques still can be guaranteed around 100 km with a block size of 10^12^. Since the finite-size effects would severely affect the theoretic maximum distance, only getting enough and reliable raw data (~10^14^) can we achieve a CV-QKD beyond 150 km. In this case, a more stable system is required.

## Discussion

We have demonstrated the longest CV-QKD experiment by controlling the excess noise. To deal with the excess noise under longer distance scenarios, we have investigated some practical approaches to enhance the SNR and reduce demands of the propagated LO power. On the one hand, we have investigated a photon-subtraction scheme that could increase the optimal *V*_*A*_ and finally improve the SNR^38^. On the other hand, the concerns of finite extinction ratio between propagated LO and quantum signal could be further reduced with frequency-shift method which was previously introduced for multichannel parallel CV-QKD^39^. While the security concerns of LO may be removed by using recent schemes with locally generated LO^40^,^41^,^42^. In addition, we note that some schemes with entangled states^43^ and noiseless amplification^44^ are also promising for long-distance CV-QKD.

Considering the finite-size effects, an efficient scheme for phase shift QKD has been proposed recently to greatly reduce the exchange information^45^. It is anticipated that this finding together with our ways of controlling excess noise will facilitate the present CV-QKD beyond 150 km. In addition, the constraint of block size and the corresponding elapsed time could be relaxed in a high-speed CV-QKD. Although several reported high-speed shot-noise-limited homodyne detectors allows us to operate at 100 MHz repetition rate^46^,^47^,^48^, the power requirement of LO for quantum measurement is orders of magnitude larger than the minimum demanding of the detector reported in this work. Therefore so far these detectors are not suitable for long-distance CV-QKD. But we believe that more efficient schemes and pioneering technological advances will bring us to the point where, in a new era of quantum information, a global quantum cryptography network is established.

## Methods

### System stability

We develop two automatic feedback control modules to enhance the stability of the CV-QKD system. (1) The modulator bias control (MBC) module. In our experiment, the light source is a narrow linewidth (1.9 kHz) and low phase noise (2 *μ*rad/rt-Hz 1 m OPD) laser producing CW coherent light at 1,550.12 nm (ITU-34). The CW light is transformed into a 2 MHz clock pulse train by a low jitter (<25 ps) digital square pulse generator and near 65 dB extinction ratio, 10 GHz *LiNbO*_3_ AM. Since the bias voltage of *LiNbO*_3_ modulator drifts with time, it will reduce the effective extinction ratio in the whole system. To stabilize the AM, we employ a MBC module to lock the working point automatically. This calibration process is used during the system initialization to maximize the extinction ratio of optical pulses, and achieved by monitoring the feedback light intensity from the output of modulator. (2) The dynamic polarization control (DPC) module. The polarization-demultiplexing of signal and LO is achieved by a DPC and polarization beam splitter at Bob’s side. The LO pulses split from the LO path at Bob’s side is detected by a PD. The analog electrical signal from the LO is fed back to bring back the State of Polarization (SOP) towards the correct SOP, which guarantees that the LO photons enter into the LO path at Bob’s side. The excess noise induced by the leaked LO photons is detailed in Supplementary S1.

### Phase compensation

We proposed an auto-correlation and reverse prediction scheme to detect the phase drift *ϕ*. At Alice’s side, Gaussian quadratures 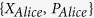 are employed to encode coherent states 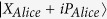, which are attenuated from a Gaussian-modulated coherent light. The quadratures can be written as


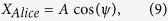



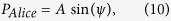


where amplitude *A* and phase *ψ* follow the Rayleigh distribution and the uniform distribution, respectively. Then the prepared coherent states will be transmitted through a Gaussian channel. Under the normal conditions, *ϕ* drifts with time slowly because of the instabilities in the AMZIs. Consequently, at Bob’s side, the corresponding homodyne measurement results are


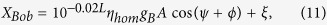



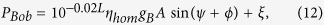


where *ξ* denotes the additive Gaussian white noise. For simplicity, only the *X* variable is considered in the following. In extremely low SNR scenarios, the measurement signal is buried in the noise,


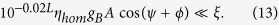


In the classical reconciliation stage, Bob announces a subset 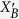 which is randomly selected from one frame. Then Alice makes a precise calculation of *ϕ* with Bob’s noisy detection results and her original frame 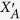. This reverse prediction procedure is finished by calculating the auto-correlation of 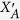 and 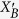,


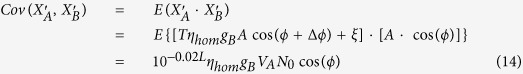


Finally, Alice maps her original data 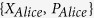 into 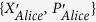 with the phase difference 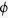, and Alice and Bob produce a raw key from 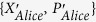 and 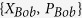. Furthermore, the security of the GMCS-QKD protocol still holds. The excess noise induced by phase compensation is detailed in Supplementary S1.

## Additional Information

**How to cite this article**: Huang, D. *et al.* Long-distance continuous-variable quantum key distribution by controlling excess noise. *Sci. Rep.* **6**, 19201; doi: 10.1038/srep19201 (2016).

## Supplementary Material

Supplementary Information
