# Supplementary material for: Long-distance continuous-variable quantum key distribution by controlling excess noise
Source: Sci Rep. 2016 Jan 13;6:19201. doi: 10.1038/srep19201 (PMC4725367; doi:10.1038/srep19201)
Supplement: Supplementary Information [file srep19201-s1.pdf]

Supplementary Material for: Long-distance continuous-variable quantum key distribution by controlling excess noise

Duan Huang,[1] Peng Huang,[1,*] Dakai Lin,[1] and Guihua Zeng[1,2,†]

[1]*State Key Laboratory of Advanced Optical Communication Systems and Networks, and Center of Quantum Information Sensing and Processing, Shanghai Jiao Tong University, Shanghai 200240, China*

[2]*College of Information Science and Technology, Northwest University, Xi'an 710127, Shaanxi, China*

This supplement contains the analysis of two major technical excess noise sources in a practical long-distance continuous-variable quantum key distribution system and some experimental details concerning the main paper.

I. ANALYSIS OF EXCESS NOISE

Excess noise is the most critical issue pertaining to long-distance continuous-variable quantum key distribution (CV-QKD). In this analysis of the excess noise, we restrict ourselves in a realistic mode, where Eve cannot tamper with the devices at Alice and Bob's boxes, but Eve would be able to exploit the internal defects at both sides, such as the inherent laser phase noise [1, 2] and the imperfect modulation [3, 4] at Alice's side, or the unbalanced homodyne detector [5] at Bob's side. Fortunately, the excess noise due to the imperfect devices mentioned above could be controlled within a tolerable limit through careful design of the experiment because these kinds of excess noise are irrelevant to the optical losses. Here, we focus on the technical excess noise in a practical long-distance experiment which is caused by the intrinsic photon loss in optical fibre.

Excess noise induced by Local Oscillator leakage. In our experiment, Alice prepares coherent light including the Local Oscillator (LO) and signal pulses, then the LO and signal pulses are transmitted together to Bob by using the time and polarization multiplexing. After demultiplexing, the LO and signal pulses interfere in a shot-noise-limited homodyne detector. The characterization of LO and signal pulses in our experiment is depicted in Fig. 1. The optical pulses are generated by an amplitude modulator (AM) with an extinction ratio R_{am} of near 65 dB as shown in Fig. 1(a). Therefore, there are always some residual photons at the interval of the LO pulses. In the time and polarization multiplexing procedure, the residual photons will leak to signal pulses limited by finite polarization extinction ratio R_{po} of near 35 dB as shown in Fig. 1(b) and Fig. 1(c). Therefore the equivalent overall extinction ratio R_e is $R_{am} + R_{po}$.

Several kinds of excess noise can be induced by LO. Let us simply describe them and assess their impacts:

(1) The excess noise $\varepsilon_{overlap}$ induced by pulses overlap between the LO (1,2,3, dark green) and the LO leakage ($1^{'}$,$2^{'}$,$3^{'}$, dark green). We have achieved 50 ns duration, 3 ns Rise (Fall) square optical pulses by external pulse modulation. The delay of the LO (1,2,3, dark green) and the LO leakage ($1^{'}$,$2^{'}$,$3^{'}$, dark green) is about 250 ns. Therefore, the overlap excess noise $\varepsilon_{overlap}$ could be neglected.

(2) The excess noise ε_{unbl} induced by the LO leakage ($1^{'}$,$2^{'}$,$3^{'}$, dark green) due to the limited common-mode rejection ratio (CMRR) in the unbalanced detection. By bending the output fiber of beam splitter (BS) in the detector, we have achieved a near 60 dB CMRR. In addition, we have achieved near 35 dB polarization extinction ratio R_{po}. This implies that the excess noise ε_{unbl} at Bob's side is near 95 dB less than the shot noise variance N_0. Accordingly, the unbalanced excess noise ε_{unbl} is negligible.

(3) The excess noise ε_{unmh} induced by unmatched LO pulse (1,2,3, dark green) in spatial mode of signal pulse ($1^{'}$,$2^{'}$,$3^{'}$, red), which would be Gaussian shaped together with signal pulse due to finite response time of the detector. In this case, $\varepsilon_{unmh}=\frac{\nu N_0}{\gamma\eta T}$, where ν is the unmatched factor, γ is the CMRR of detector, η is the quantum efficiency of detector, and T is the transmission efficiency. We have achieved a 10 ps time-multiplexing precision by a manual variable optical delay line (MVODL, see Fig.1 in the main body), which features a readout scale resolution of 0.05 mm. We find that the unmatched factor υ_{el} is near -50 dB. This implies that the excess noise ε_{unmh} at Bob's side is near 110 dB less than the shot noise variance N_0 at Bob's side. Therefore, the unbalanced excess noise ε_{unmh} could be neglected.

(4) The excess noise ε_{flu} induced by LO fluctuations. The ε_{flu} is associated with the stability of laser, pulse modulation and fibre channel. It is monitored in real time by a photodetector (PD) at Bob's side. We note that the excess noise ε_{flu} due to the imperfect laser and pulse modulation is monitored by a PD at Alice's side, and can be subtracted based on the above realistic assumptions.

(5) The excess noise ε_{LE} induced by residual photons (1,2,3, cyan) in the interval of LO pulses. Controlling of the excess noise ε_{LE} imposes a great challenge for the long-distance CV-QKD because it is difficult to

*Electronic address: huang.peng@sjtu.edu.cn
†Electronic address: ghzeng@sjtu.edu.cn

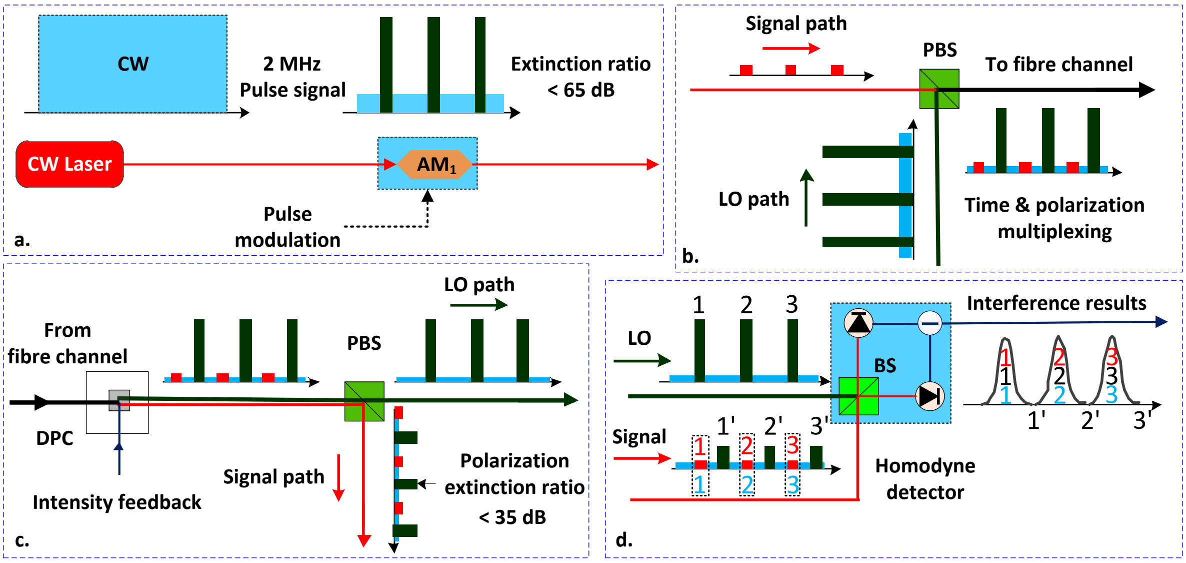

FIG. 1: **The characterization of LO and signal pulses** (**a**) Pulse modulation. A continuous-wave (CW) coherent light is transformed into a 2 MHz clock pulse train by a high extinction ratio Amplitude Modulator (AM). (**b**) Time and polarization multiplexing. The LO and the delayed signal pulses are multiplexed with orthogonal polarization using a polarizing beamsplitter (PBS). (**c**) Demultiplexing. The LO and signal pulses are demultiplexed with a PBS and a dynamic polarization controller (DPC). (**d**) Balanced homodyne detection. The demultiplexed LO (1,2,3, dark green) and signal pulses ($1^{'}$,$2^{'}$,$3^{'}$, red) interfere on a shot-noise-limited homodyne detector. The pulses ($1^{'}$,$2^{'}$,$3^{'}$, dark green) leaked from LO pulses due to the limited polarization extinction ratio will be divided equally in the beamsplitter (BS) and removed in the interference results. The leakage photons (1,2,3, cyan) leaked from the interval of LO pulses due to the limited overall extinction ratio will be interfered with LO, and it has become a major source of excess noise in long-distance CV-QKD.

completely remove the residual photons in an optical system with a finite extinction ratio R_e. Moreover, the leaked photons and the signal photons will simultaneously interfere with LO, and the excess noise ε_{LE} and V_A would be of the same order of magnitude even under one photon leakage condition. To control the excess noise ε_{LE}, one has to decrease the LO power at Alice's side $\langle \hat{N}_{LO}^{Alice} \rangle$. However, homodyne detection of coherent states under the shot noise limit requires sufficient LO power of $\langle \hat{N}_{LO}^{Bob} \rangle (\approx 10^{-0.02L} \langle \hat{N}_{LO}^{Alice} \rangle)$ at Bob's side. This becomes one of major constraints in the long-distance CV-QKD.

In the following, we derive the excess noise ε_{LE}, and then estimate the order of magnitude of ε_{LE} in a 100∼150 km CV-QKD experiment. The quantum state received at Bob's side can be denoted by the quadratures (X, P) which satisfy

$$\begin{aligned} X &= \sqrt{\eta T}(X_A + \delta X_c) + \delta X_A, \\ P &= \sqrt{\eta T}(P_A + \delta P_c) + \delta P_A, \end{aligned} \qquad (1)$$

where X_A and P_A are the modulated values with variance $\langle X_A^2 \rangle = \langle P_A^2 \rangle = V_A = 2\langle \hat{N}_{sig} \rangle$, and $\delta X_A(\delta P_A)$ and $\delta X_c(\delta P_c)$ are originated from the shot noise and channel excess noise, which satisfy $\langle \delta X_A^2 \rangle = \langle \delta P_A^2 \rangle = 1$ and $\langle \delta X_c^2 \rangle = \langle \delta P_c^2 \rangle = \varepsilon_c$ in shot noise units, respectively.

The average number of residual photons (1,2,3, cyan) leaked from the interval of LO pulses to signal pulses is

$$\langle \hat{N}_{LE} \rangle = \frac{\langle \hat{N}_{LO}^{Alice} \rangle}{R_e}. \qquad (2)$$

Therefore, the interference results in Fig. 1(d) is given by

$$\begin{aligned} X' &= \sqrt{\eta T}(X'_A + \delta X_c) + \delta X_A, \\ P' &= \sqrt{\eta T}(P'_A + \delta P_c) + \delta P_A, \end{aligned} \qquad (3)$$

where X'_A and P'_A are the modified values with variance of $\langle X_A'^2 \rangle = \langle P_A'^2 \rangle = V_A + 2\langle \hat{N}_{LE} \rangle$. The excess noise induced by the LO leakage can be regarded as an extra excess noise. Then the measurement results can be rewritten as

$$\begin{aligned} X' &= \sqrt{\eta T}(X_A + \delta X_e + \delta X_c) + \delta X_A, \\ P' &= \sqrt{\eta T}(P_A + \delta P_e + \delta P_c) + \delta P_A, \end{aligned} \qquad (4)$$

where δX_e and δP_e are the extra excess noise. Therefore the excess noise ε_{LE} can be expressed as

$$\varepsilon_{LE} = \langle \delta X_e^2 \rangle = \langle \delta P_e^2 \rangle = 2\langle \hat{N}_{LE} \rangle = \frac{2\langle \hat{N}_{LO}^{Alice} \rangle}{R_e}. \qquad (5)$$

According to Eq. (5), we estimate the order of magnitude of ε_{LE} and study the impact of the excess noise ε_{LE} on our long-distance experiment with consideration of finite-size effects presented in [6, 7]. With a typical

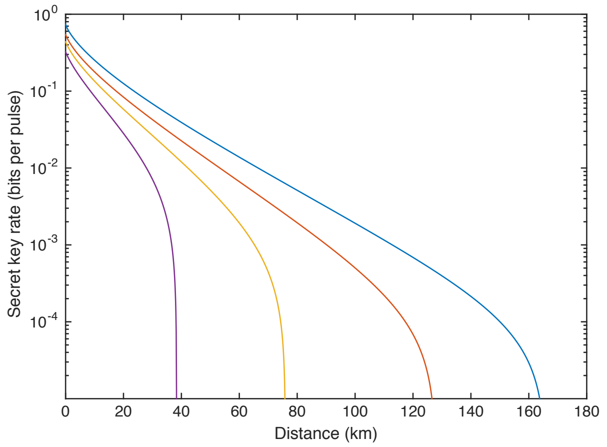

FIG. 2: **The secret key rate under general collective attack for different excess noise ε_{LE}.** The curves from top to bottom correspond to $\varepsilon_{LE} = 0.01, 0.05, 0.08, 0.12$.

$\langle \hat{N}_{LO}^{Alice} \rangle$ of 10^8 photons/pulse and an extinction ratio R_e of 100 dB, we can effectively control the excess noise ε_{LE} in the order of 0.01. In Fig. 2, The secret key rate respect to secure distance are simulated for different values of excess noise ε_{LE} with typical parameters of $V_A = 4$, $\beta = 0.956$, $\varepsilon_c = 0.002$, $\eta_{hom} = 0.6$, $v_{el} = 0.01$, block size of 10^{12} and security parameter ϵ of 10^{-10}. It is clear that the increase of the excess noise ε_{LE} will significantly restrict the secure distance. Especially, $\varepsilon_{LE} = 0.01$ is a tolerable value in our 100∼150 km CV-QKD experiment.

Excess noise due to inaccuracy of phase compensation. Here we show the derivation of the excess noise due to inaccuracy of the phase compensation. Under the perfect phase compensation, we denote the quadratures of the output mode of quantum channel as (X_B^t, P_B^t). The quadratures without phase compensation can be expressed as

$$\begin{aligned} X_B &= X_B^t + X_B^{pd}, \\ P_B &= P_B^t + P_B^{pd}, \end{aligned} \tag{6}$$

where (X_B^{pd}, P_B^{pd}) are the added quadratures arising from the phase drift ϕ, which satisfy $X_B^{pa} = |\alpha| \cos\phi$ and $P_B^{pa} = |\alpha| \sin\phi$. After receiving the quantum state, Bob will rectify the phase of the state with the phase compensation algorithm, which can be equivalently denoted as

$$\begin{aligned} X &= X_B - X_B^{pc} = X_B^t + (X_B^{pd} - X_B^{pc}), \\ P &= P_B - P_B^{pc} = P_B^t + (P_B^{pd} - P_B^{pc}), \end{aligned} \tag{7}$$

where (X_B^{pc}, P_B^{pc}) are the subtracted quadratures from the phase compensation. They satisfy $X_B^{pc} = |\alpha| \cos\phi'$ and $P_B^{pc} = |\alpha| \sin\phi'$, where ϕ' is the phase angle in the compensation algorithm. If the phase compensation is perfect, one would get $\phi = \phi'$ and $(X_B^{pd}, P_B^{pd}) = (X_B^{pc}, P_B^{pc})$. In this case, the excess noise ε_{phase} would be eliminated. Thus one obtains

$$X = X_B^t, P = P_B^t. \tag{8}$$

However, because of the difficulty of phase compensation at low SNR, the phase angle ϕ' in the compensation algorithm is not equal to the drifted phase ϕ. Defining the inaccuracy of phase compensation as $\delta\theta = |\phi - \phi'|$, the inaccuracy of phase compensation can be regarded as a phase shift operation $U(\delta\theta) = \exp(i\delta\theta a^\dagger a)$ on Bob's measurement results, and $\delta\theta$ follows a probability of $p(\delta\theta)$. When the quantum channel between Alice and Bob is characterized by transmittance T and channel excess noise ε_c, the covariance matrix of the state ρ_{AB} shared by Alice and Bob can be expressed as

$$\gamma_{AB} = \begin{pmatrix} V\mathbb{1}_2 & W\sqrt{T}\sigma_z \\ W\sqrt{T}\sigma_z & T(V+\chi_{tot})\mathbb{1}_2 \end{pmatrix}, \tag{9}$$

here $\mathbb{1}_2$ is the identity matrix, $W = \sqrt{V^2 - 1}$ with $V = V_A + 1$, σ_z=diag(1,-1), and $\chi_{tot} = \frac{1+\nu_{el}}{T\eta} - 1 + \varepsilon_c$. The covariance matrix of the output of the state ρ_{AB} after the phase shift operation $U(\delta\theta)$ is then derived as

$$\gamma(\delta\theta) = \begin{pmatrix} V\mathbb{1}_2 & \mathbb{W} \\ \mathbb{W} & T(V+\chi_{tot})\mathbb{1}_2 \end{pmatrix}, \tag{10}$$

where

$$\mathbb{W} = \begin{pmatrix} W\sqrt{T}\cos\delta\theta & -W\sqrt{T}\sin\delta\theta \\ -W\sqrt{T}\sin\delta\theta & -W\sqrt{T}\cos\delta\theta \end{pmatrix}. \tag{11}$$

Then the state affected by the phase noise is a classical mixture of states with random phase shifts

$$\rho'_{AB} = \int (\mathbb{1}_A \otimes U_B(\delta\theta))\rho_{AB}(\mathbb{1}_A \otimes U_B(\delta\theta))p(\delta\theta)d\delta\theta, \tag{12}$$

which corresponds to the covariance matrix

$$\gamma'_{AB} = \begin{pmatrix} V\mathbb{1}_2 & \sqrt{\kappa T}W\sigma_z \\ \sqrt{\kappa T}W\sigma_z & T(V+\chi_{tot})\mathbb{1}_2 \end{pmatrix}, \tag{13}$$

where we assumed that the distribution $\delta\theta$ is symmetric. More specifically, $\delta\theta$ satisfies $\int p(\delta\theta)\sin\delta\theta d\delta\theta = 0$, and $\kappa = (\int p(\delta\theta)\cos\delta\theta d\delta\theta)^2 = (E[\cos\delta\theta])^2$, where $E[X]$ denotes the expectation of the random variable X. Therefore, the equivalent transmittance T_κ and the excess noise ε_c^κ of quantum channel after phase compensation can be expressed as

$$\begin{aligned} T_\kappa &= \kappa T, \\ \varepsilon_c^\kappa &= [\varepsilon_c + (1-\kappa)(V-1)]/\kappa. \end{aligned} \tag{14}$$

Subsequently, the excess noise ε_{phase} due to the imperfect phase compensation is given by,

$$\varepsilon_{phase} = \varepsilon_c^\kappa - \varepsilon_c = (1-\kappa)(\varepsilon_c + V_A)/\kappa. \tag{15}$$

II. EXPERIMENTAL DETAILS

Extinction ratio of the optical system. As discussed above, the excess noise ε_{LE} is caused by the limited extinction ratio of the optical system. By definition, the extinction ratio R_e is the ratio of one level and zero level of digital optical pulses in telecommunications, and it can be given by,

$$R_e = \frac{P_1}{P_0}, \tag{16}$$

where P_1 is the optical power of one level which is generated when the laser diode is on, and P_0 is the optical power of zero level which is generated when the laser diode is off. In most direct modulation, the laser must be biased so that P_0 is in the vicinity of the laser threshold, meaning P_0>0. The extinction ratio is limited by the modulation depth. Moreover, the direct modulation may result in large frequency drifts (chirp). In comparison, the external modulation has remarkable advantages [8, 9], especially for narrow linewidth, low noise, high extinction ratio, and wideband optical signal generation. Therefore, the external modulation is used for pulse generation in our experiment, and we prefer to restrict ourselves in such mode for the analysis of the LO leakage.

The main aim of employing external pulse modulation in long-distance CV-QKD is to enhance the extinction ratio R_e so that the LO leakage is reduced. However, the extinction ratio in our experiment is mainly limited by the $LiNbO_3$ AM (< 65 dB). We note that the optical switch features wide modulation bandwidth and high extinction ratio (as high as 75 dB by the fine tuning subsystem) in optical pulse generation [10]. Thus, further improvement of the secure distance in CV-QKD experiment could be achieved with the optical switch.

Shot-noise-limited homodyne detection With a typical LO power $\langle \hat{N}_{LO}^{Alice} \rangle$ of 10^8 photons/pulse and a 150 km standard telecom fibre, the effective LO power $\langle \hat{N}_{LO}^{Bob} \rangle$ for homodyne detection is about 10^5 photons/pulse. To achieve a shot-noise-limited homodyne detection, the electronic noise of the detector must be reduced under such extreme conditions. We employed the field-effect transistors (FETs) in our first-stage electronic design. The FET temperature noise model [11] may be expressed as,

$$\upsilon_{\mathrm{FET},el} = |\mathbf{Z}_{\mathrm{FET},in} - \mathbf{Z}_{\mathrm{FET},out}|^2 \cdot \frac{4k_B T_{\mathrm{FET}} B}{R_{\mathrm{FET}}}, \tag{17}$$

where k_B is the Boltzman's constant, T_{FET} is the temperature of the FETs in the detector, B is the noise bandwidth, R_{FET} is the equivalent resistor of FETs, and $\mathbf{Z}$ is the inverse admittance matrix of the circuit. The obvious way to suppress $\upsilon_{\mathrm{FET},el}$ is to reduce the temperature (−50°C in our case). A similar cooling FET detector has been used in the photon-number-resolving detection [12]. In addition, lower noise FET, more complex electronic circuit design around the FET, and additional filter can also reduce $\upsilon_{\mathrm{FET},el}$. Actually, a practical low noise detector is very difficult because of the stray capacitance on the circuit board, the noise performance of resistors, leakage and dark current and so on. Since small variations of the capacitance and resistance would result in larger υ_{el} and lower bandwidth, the process of controlling electronic noise is imprecise. Though the noise of the

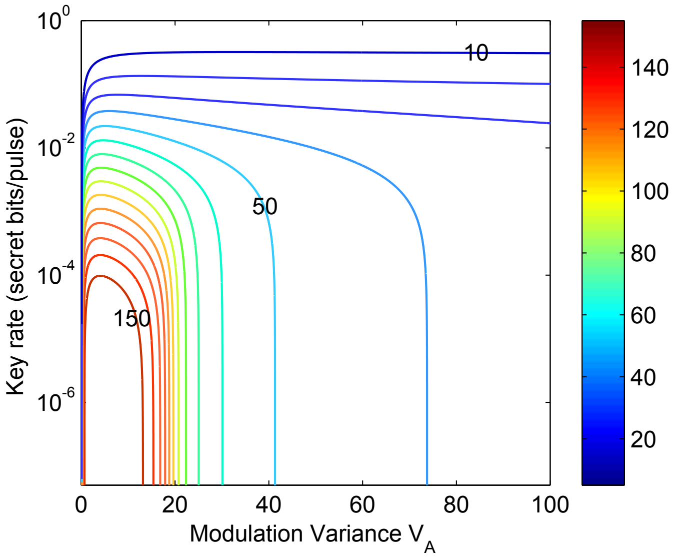

FIG. 3: **Key rate as a function of modulation variance V_A for various distances under the assumption of collective attacks.** $\eta = 0.6$, $\upsilon_{el} = 0.01$, $\varepsilon = 0.01$, $\alpha_{fibre} = 0.2$ dB/km, and $\beta = 95.6\%$. From top to bottom, the distance increases by 10 km. υ_{el}, ε and V_A is normalized in shot noise variance N_0.

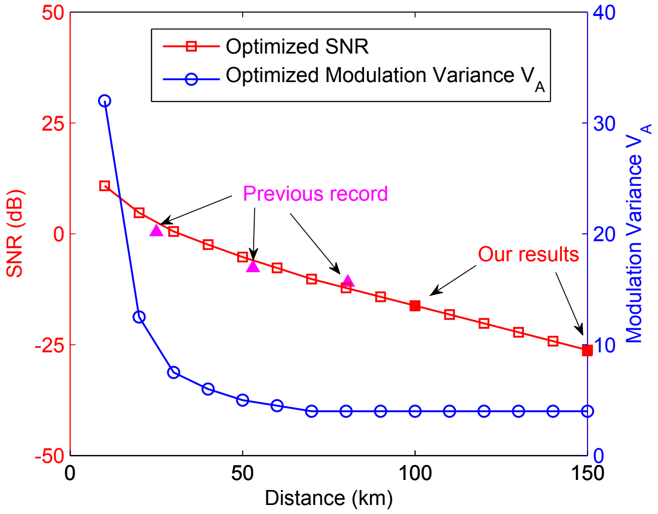

FIG. 4: **Optimized modulation variance V_A and the corresponding optimized SNR as a function of distance under the assumption of collective attacks.** $\eta = 0.6$, $\upsilon_{el} = 0.01$, $\varepsilon = 0.01$, $\alpha_{fibre} = 0.2$ dB/km, and $\beta = 95.6\%$.

TABLE I: Code rates, channel efficiencies and SNR thresholds on the AWGNC of the MET-LDPC codes.

R	β	SNR
0.5	95.03%	1.0739
0.5	94.35%	1.0851
0.5	95.85%	0.1556
0.05	95.8%	0.075
0.02	96.9%	0.029

first stage $\upsilon_{\mathrm{FET},el}$ completely dominates the noise of the entire system υ_{el}, one has to keep in mind that the low noise shaping amplifiers and the current-feedback amplifiers are also very important in the high fidelity signal processing. Further improvement of noise performance of the detector could be achieved by reducing the temperature to several K and optimizing the electronic circuit.

Optimization of modulation variance. We mainly focus on the optimization of parameter V_A and the corresponding threshold of SNR (SNR_{th}) so as to employ an available LDPC code in the classical reconciliation. The relationship of V_A and SNR is given by,

$$\mathrm{SNR} = \frac{10^{-0.02L}\eta_{hom}V_A N_0}{N_0 + 10^{-0.02L}\eta_{hom}\varepsilon_{tot} + \upsilon_{el}} > \mathrm{SNR}_{th}, \quad (18)$$

where ε_{tot} is the total excess noise of the system. The key rate as a function of V_A for various distances is shown in Fig. 3. It is clear that an optimization of parameter V_A can effectively improve the secure distance and key rate. To achieve a 150 km CV-QKD experiment, the maximum V_A should be less than 15. Fig. 4 shows the optimized modulation variance V_A and the corresponding SNR as a function of distance. The optimal modulation variance V_A appears to be starting to stabilize around 4 from the distance of 100 km, and the corresponding SNR decreases linearly with the channel loss. The SNRs of previous record show a 0.17 dB at 53 km and 0.08 dB at 80.5 km at Bob's side [7]. In our experiment, the actual value of SNR at Bob's side is about 0.024 at 100 km and 0.0024 at 150 km, which is lower by more than an order of magnitude compared with the previous record.

Error correction. In our experiment, an ECC with high-efficiency and low SNR threshold is utilized in the reconciliation procedure. Such code is designed by combining the Multiedge-type low density parity check (MET-LDPC) code [13] and the repetition scheme [14]. Generally, the MET-LDPC code exhibits better performance than the standard LDPC code. Its efficiency $\beta(s)$ is associated with the Gaussian channel capacity $C(s)$, i.e.,

$$\beta(s) = \frac{R}{C(s)} = \frac{R}{\frac{1}{2}\log_2(1+s)}, \quad (19)$$

where R is the code rate, and s denotes the SNR. Using the technique of repetition scheme with a repetition factor of k, a code exhibiting a rate R and an efficiency $\beta(s)$

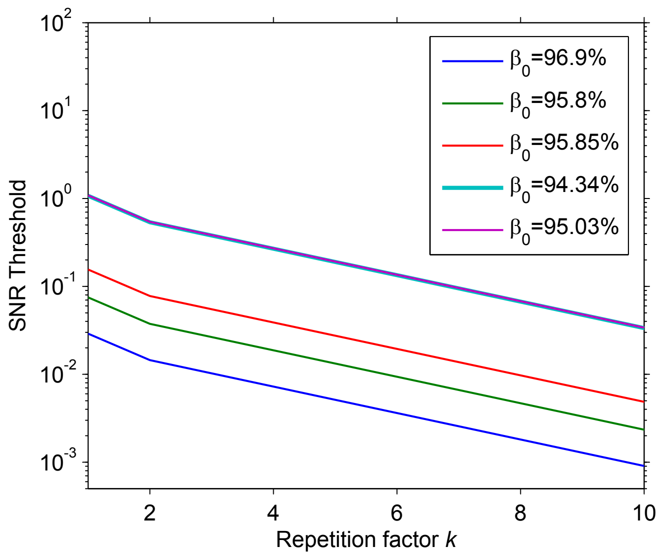

FIG. 5: **SNR threshold as a function of repetition factor k for different codes in Table I.**

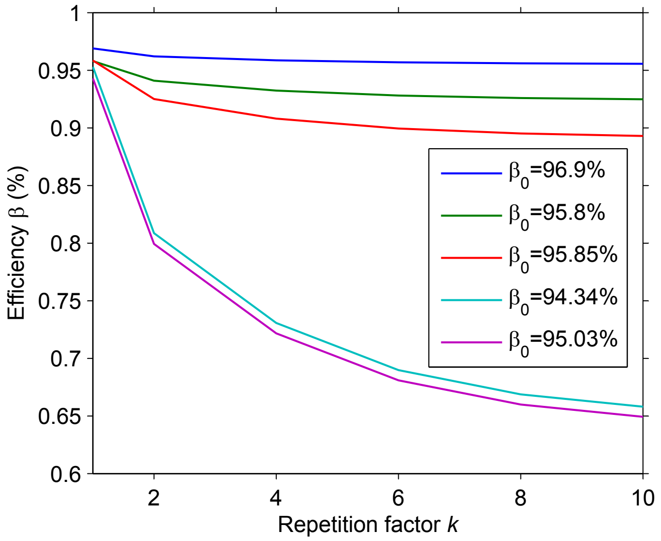

FIG. 6: **Efficiency of error correction as a function of repetition factor k for different codes in Table I.**

with a threshold of SNR s may be converted into a new code which exhibits a rate $R' = R/k$, a SNR $s' = s/k$, and an efficiency

$$\beta'(s/k) = \beta(s)\frac{\frac{R/k}{\log(1+s/k)}}{\frac{R}{\log(1+s)}} = \beta(s)\frac{\log(1+s)}{k\log(1+s/k)}. \quad (20)$$

Eq.(20) shows that we can achieve a new code with high efficiency and lower threshold.

For clarity, we exemplify the above way by utilizing some typical MET-LDPC codes for white Gaussian noise channel (AWGNC)[13, 15]. These codes are listed in Tab. I. As we can see from Fig. 5, the repetition scheme can help us to achieve an extremely low SNR threshold.

Then we compute the efficiencies with the scheme on the AWGNC for different SNRs in Fig. 6. It shows that the repetition scheme allows a low efficiency loss only for specific codes with low initial SNR threshold (SNR_0<0.2 in our case) though all of the codes offer seductively high initial efficiency (∼95%). Based on the original codes in the Tab. I, we developed a MET-LDPC code with good efficiency (>95%) and low SNR threshold (∼0.001), which is available for the 150 km CV-QKD experiment.

[1] Lodewyck, J., Debuisschert, T., Tualle-Brouri, R., & Grangier, P. (2005). Controlling excess noise in fiber-optics continuous-variable quantum key distribution. *Phys. Rev. A* **72**(5), 050303.

[2] Huang, P., He, G. Q., & Zeng, G. H., Bound on Noise of Coherent Source for Secure Continuous-Variable Quantum Key Distribution. *Int. J. Theor. Phys.* **52**, 1572-1582 (2013).

[3] Jouguet, P., Kunz-Jacques, S., Diamanti, E., & Leverrier, A. Analysis of imperfections in practical continuous-variable quantum key distribution. *Phys. Rev. A* **86**(3), 032309 (2012).

[4] Shen, Y., Peng, X., Yang, J., & Guo, H. (2011). Continuous-variable quantum key distribution with Gaussian source noise. *Phys. Rev. A* **83**(5), 052304 (2011).

[5] Chi, Y. M. *et al.* A balanced homodyne detector for high-rate Gaussian-modulated coherent-state quantum key distribution. *New J. Phys.* **13**, 013003 (2011).

[6] Leverrier, A., Grosshans, F., & Grangier, P., Finite-size analysis of a continuous-variable quantum key distribution. *Phys. Rev. A* **81**, 062343 (2010).

[7] Jouguet, P., Kunz-Jacques, S., Leverrier, A., Grangier, P. & Diamanti, E. Experimental demonstration of long-distance continuous-variable quantum key distribution. *Nature Photon.* **7**, 378-381 (2013).

[8] Cox III, C. H., Betts, G., & Johnson, L. M. An analytic and experimental comparison of direct and external modulation in analog fiber-optic links. Microwave Theory and Techniques, *IEEE Transactions on Microwave Theory and Techniques*, **38**(5), 501-509 (1990).

[9] Peucheret, C. (2009). Direct and external modulation of light.

[10] Thorlabs: High-Speed Optical Shutter/Switch.

[11] Garcia, M., Stenarson, J., Zirath, H., & Angelov, I., A direct extraction formula for the FET temperature noise model. *Microwave Opt. Techn. Lett.*, **16**(4), 208-212 (1997).

[12] Fujiwara, M., & Sasaki, M., Photon-number-resolving detection at a telecommunications wavelength with a charge-integration photon detector. *Opt. Lett.* **31**(6), 691-693 (2006).

[13] Richardson, T. & Urbanke, R. Multi-edge type LDPC codes,In Workshop honoring Prof. Bob McEliece on his 60th birthday, California Institute of Technology, *Pasadena, California, USA* (2002).

[14] Leverrier, A., & Grangier, P. Continuous-variable quantum key distribution protocols with a discrete modulation. e-print arXiv:1002.4083 (2010).

[15] Jouguet, P., Kunz-Jacques, S. & Leverrier, A. Long-distance continuous-variable quantum key distribution with a Gaussian modulation. *Phys. Rev. A* **84**, 062317 (2011).
